# Effects of chia seed (*Salvia hispanica L.*) supplementation on cardiometabolic health in overweight subjects: a systematic review and meta-analysis of RCTs

**DOI:** 10.1186/s12986-024-00847-3

**Published:** 2024-09-16

**Authors:** Mehdi Karimi, Samira Pirzad, Niyousha Shirsalimi, Sajad Ahmadizad, Seyyed Mohammad Hashemi, Shaghayegh Karami, Kimia Kazemi, Erfan Shahir-Roudi, Anita Aminzadeh

**Affiliations:** 1 Bogomolets National Medical University (NMU), Kyiv, Ukraine; 2 Faculty of Medicine, Islamic Azad University, Tehran Medical Sciences Branch (IAUTMU), Tehran, Iran; 3 Faculty of Medicine, Hamadan University of Medical Science (UMSHA), Hamadan, Iran; 4 Department of Biological Sciences in Sport, Faculty of Sport Sciences and Health, Shahid Beheshti University, Tehran, Iran; 5 Student Research Committee, Faculty of Medicine, Hormozgan University of Medical Sciences, Bandar Abbas, Iran; 6 School of Medicine, Tehran University of Medical Sciences (TUMS), Tehran, Iran; 7 Department of Food Science and Technology, Ayatollah Amoli Branch, Islamic Azad University, Amol, Iran; 8 Student Research Committee, School of Public Health, Shahroud University of Medical Sciences, Shahroud, Iran

**Keywords:** Chia seed, Saliva, Cardiometabolic, Metabolic, Overweight, Lipid, Glycemic

## Abstract

**Background:**

Obesity is a significant public health issue associated with various chronic diseases. Research has indicated that chia seeds have the potential to improve cardiometabolic health. However, due to the diversity of research and inconsistencies in study design, further investigation is needed to fully understand their clinical effects on overweight individuals. This review aims to comprehensively analyze the available evidence on the effects of chia seeds on cardiometabolic indices in overweight populations through a meta-analysis.

**Methods:**

A comprehensive literature search was performed across PubMed, Web of Science, Scopus, and Embase databases from their inception until 01-03-2024 to identify randomized controlled trials (RCTs) evaluating the effect of chia on cardiometabolic indices in overweight subjects. The search strategy incorporated both Medical Subject Headings (MeSH). Following the screening, ten RCTs were finally included. The data, including subject characteristics, study design, and changes in serum biomarkers, were extracted and analyzed using Stata software version 18.

**Results:**

The meta-analysis results reveal that chia supplementation no significant changes in lipid profile, including triglycerides (TG) (MD: − 5.80 mg/dL, p = 0.47), total cholesterol (TC) (MD: − 0.29 mg/dL, p = 0.95), high-density lipoprotein (HDL) (MD: 1.53 mg/dL, p = 0.33), and low-density lipoprotein (LDL) (MD: 0.63 mg/dL, p = 0.88). Similarity fasting blood glucose (FBG) (MD: − 0.03 mg/dL, p = 0.98), hemoglobin A1c (HbA1c) (MD: − 0.13%, p = 0.13), and insulin levels (MD: 0.45 µIU/mL, p = 0.78). However, chia seed supplementation was associated with a significant reduction in C-reactive protein (CRP) (MD: − 1.18 mg/L, p < 0.0001), but no significant changes were observed in interleukin-6 (IL-6) (MD: − 0.15, p = 0.70) or tumor necrosis factor-alpha (TNF-α) (MD: 0.03, p = 0.91). There was no significant effect on body mass index (BMI) (MD: 0.1 kg/m^2^, p = 0.91), but a significant reduction in waist circumference (WC) (MD: − 2.82 cm, p < 0.001) was noted. Additionally, chia seed supplementation resulted in a significant reduction in systolic blood pressure (BP) (MD: − 3.27 mmHg, p = 0.03), though diastolic BP changes were non-significant (MD: − 2.69 mmHg, p = 0.09). The studies showed low to moderate heterogeneity in outcome measures, with I^2^ < 50%.

**Conclusion:**

Chia seed supplementation does not significantly impact most lipid profile parameters and glycemic markers. However, it shows potential benefits in reducing WC, BP, and CRP. While chia seeds can be a valuable addition to cardiometabolic health management, they should be part of a broader health strategy that includes a balanced diet, exercise, and lifestyle modifications for optimal results.

**Supplementary Information:**

The online version contains supplementary material available at 10.1186/s12986-024-00847-3.

## Introduction

In recent decades, the rates of overweight and obesity have significantly increased, becoming major public health concerns [1]. Obesity, characterized by excessive fat accumulation that poses health risks, affects over 650 million people worldwide, according to the World Health Organization (WHO). This rising prevalence of obesity signifies a widespread pandemic with significant health implications [2]. Obesity is linked to a higher risk of developing other conditions such as type 2 diabetes mellitus (T2DM), dyslipidemia, metabolic syndrome (MetS), and cardiovascular diseases (CVD) [3]. Cardiometabolic conditions remain the leading causes of death and illness globally, according to a 2000–2019 assessment [4]. Controlling anthropometric measures, blood pressure, glycemic indices, lipid profiles, and inflammatory markers is crucial for maintaining health [5]. These markers are strongly linked to a higher risk of chronic illnesses like obesity [6]. The interest in developing effective therapies to enhance these health indicators is increasing. Dietary adjustments are highlighted as crucial in preventing metabolic diseases, supported by experimental and epidemiological research [7, 8]. There is a growing demand for nutritionally balanced herbal products, with chia seed supplementation gaining particular interest [9, 10].

*Salvia hispanica*, commonly known as chia seeds, is an herbaceous plant in the *Lamiaceae* family. It is rich in protein, omega-3 fatty acids, and both soluble and insoluble fibers. Traditionally used as a dietary and therapeutic resource, chia seeds have recently garnered interest for their unique chemical profile [11, 12]. They may aid in weight reduction and address obesity-related challenges, offering potential health benefits. Additionally, chia seeds possess anti-inflammatory and antioxidant properties, further contributing to their therapeutic potential [11–13].

Although research specifically targeting overweight patients is limited, chia seeds are thought to offer several cardiometabolic benefits. According to recent research, chia seeds may improve lipid profile, glycemic markers, antioxidant properties, blood pressure (BP) regulation, and weight management [14, 15]. Over the last two decades, research has indicated that chia seeds have beneficial outcomes regarding insulin resistance, lipid abnormality, glucose tolerance, and obesity [16–20]. According to recent research, chia seeds may improve blood lipid profiles because of their numerous beneficial components; this is due to their ability to lower blood glucose levels, fight against microbes, reduce blood pressure, and stimulate the immune system [21]. Nevertheless, these studies exhibit significant disparities in the number of participants, individual characteristics, and sample composition, with a greater emphasis on animal research rather than human studies.

Despite the increasing number of studies examining the effects of chia seed supplementation on health markers related to overweight and cardiometabolic conditions, there remains a significant gap in the consistent and comprehensive analysis of these effects in human populations. Existing research shows varied results across different sample sizes and characteristics. Therefore, this study aims to systematically review and evaluate the cardiometabolic effects of chia seeds on overweight individuals from diverse populations, specifically focusing on their impact on cardiometabolic factors.

## Methods

### Protocol and registration

This systematic review and meta-analysis were conducted following the guidelines of the Preferred Reporting Items for Systematic Reviews and Meta-Analyses (PRISMA) statement to ensure transparency and comprehensive reporting [22]. The research protocol was registered with the International Prospective Register of Systematic Reviews (PROSPERO) under the registration number CRD42024538150 to facilitate methodological rigor and reproducibility.

### Search strategy

This systematic review and meta-analysis involved a comprehensive search of several scientific databases, including PubMed, Web of Science, Scopus, Embase, and Google Scholar, from inception until March 2024. The objective was to identify RCTs evaluating the effect of Chia (*Salvia hispanica L.*) on cardiometabolic indices in overweight patients. The search strategy utilized a combination of relevant MeSH (Medical Subject Headings) terms and non-MeSH phrases, employing Boolean operators (AND, OR) by searching [Title/Abstract] without any time restrictions. The search was limited to articles published in English. Additionally, the reference lists of all relevant articles were manually searched to identify any potentially eligible studies, including grey literature, that may have been missed during the database searches.

### Inclusion and exclusion criteria

We included original RCTs that investigated the impact of the Chia (intervention) on cardiometabolic indices (outcome) among overweight healthy subjects (population). Furthermore, our inclusion criteria embrace studies exclusively in English. Our exclusion criteria meticulously refined the focus of our research by excluding various study designs such as cross-sectional, case series, case–control, and cohort studies, as well as systematic reviews, meta-analyses, abstracts, and letters to the editor. Additionally, we excluded in vitro, in vivo, and animal studies to streamline our analysis of human subjects. Furthermore, articles not published in English were excluded to ensure consistency in data interpretation and facilitate comprehension. These stringent criteria enhanced the validity and applicability of our research findings to the target population of overweight individuals undergoing Chia supplementation. Guided by the Patient, Intervention, Comparison, and Outcome (PICO) model [23], our research question was formulated (Table 1).

**Table 1:** The population, intervention, comparison, outcome, and study design (PICO) criteria

Domain	Selection criteria
Participants	Overweight subjects (healthy and patient)
Intervention group	Supplementation with Chia
Comparison group	Placebo, Control
Outcomes	Cardiometabolic indices (BMI, WC, BP, FBG, HbA1C, Insulin, LDL, HDL, TG, TC, CRP, IL-6, TNF-α)

### Data extraction

The data from the included studies were screened and extracted independently by two reviewers (*E.SH.R.* and *A.A.*) based on the study inclusion criteria explained above, and any disagreements during data extraction were resolved through discussion between the two reviewers, if consensus could not be reached, a third reviewer (*M.K.*) was consulted. Extracted data including the name of the first author, year of publication, participants characteristics (mean age, gender), study design, sample sizes, country setting, and mean ± standard deviation (SD) of changes in cardiometabolic indices, including anthropometric indices (BMI, WC), blood pressure (systolic and diastolic), glycemic markers (FBG, Insulin, HbA1c,), lipid profile (LDL, HDL, TG, TC), and inflammatory markers (CRP, IL-6, TNF-α) in each group of intervention and control.

### Quality assessment

**Fig. 1:**
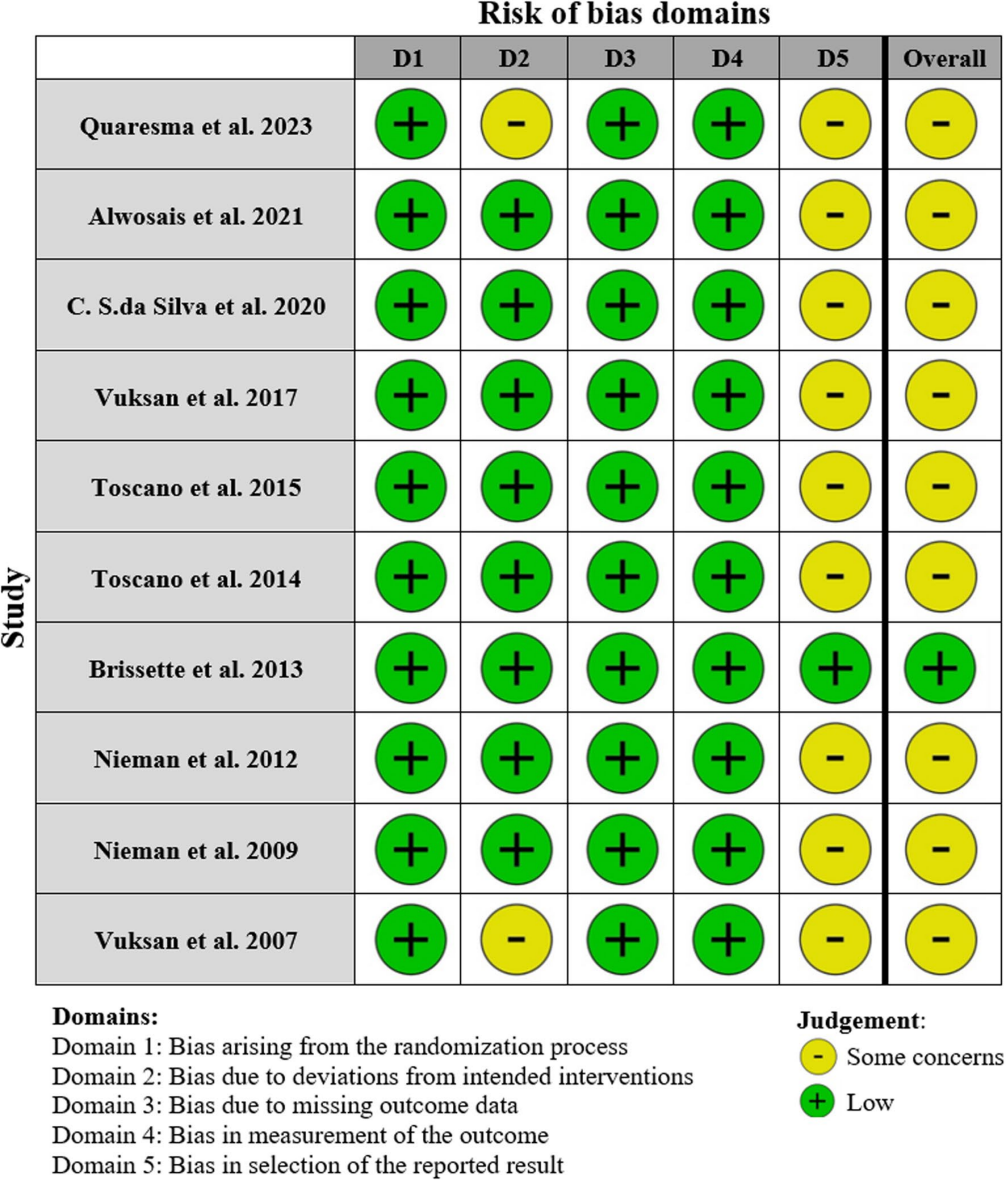
Cochrane risk of bias assessment (RoB2)

Two researchers (*S.P. & E.SH.R.*) meticulously reviewed the methodological quality of each included study using the Revised Cochrane risk-of-bias instrument for randomized trials (RoB-2). Any disagreements were resolved through discussion between the two reviewers, and if consensus could not be reached, a third reviewer (*M.K.*) was consulted. RoB-2 is specifically designed to assess the likelihood of bias in randomized controlled trials (RCTs), ensuring a rigorous evaluation process. The RoB2 tool examines various domains of bias, including bias arising from the randomization process, deviations from intended interventions, missing outcome data, measurement of the outcome, and selection of the reported result [16] (see Fig. 1).

### Statistical analyses

The effect size for the outcomes was evaluated using the mean difference (MD) along with 95% confidence intervals (CI) to quantify the impact of chia seed supplementation on cardiometabolic indices [24]. When studies provided only baseline and end-of-trial means without reporting standard deviations (SD) for changes, the SD for the net change was estimated using the Follmann method, assuming a correlation coefficient (R) 0.5. If the Standard Error (SE) was provided, the SD was calculated using the formula SD = SE × sqrt(n), where n represents the sample size in each group [25]. Heterogeneity among studies was assessed using the I^2^ statistic and Cochrane’s Q-test, with I^2^ values greater than 50% and a P-value of less than 0.05, indicating substantial heterogeneity [26]. A random-effects model was applied to account for potential variability across studies. For trials assessing multiple interventions, each was analyzed as a separate effect size to ensure accuracy. Sensitivity analysis was conducted to test the robustness of the findings, while subgroup analyses explored the influence of specific study characteristics and excluded studies with a high risk of bias. Begg’s and Egger’s tests were performed to assess publication bias, and funnel plots were used to visually inspect for asymmetry. All statistical analyses were conducted using Stata software version 18.

## Results

### Study selection

**Fig. 2:**
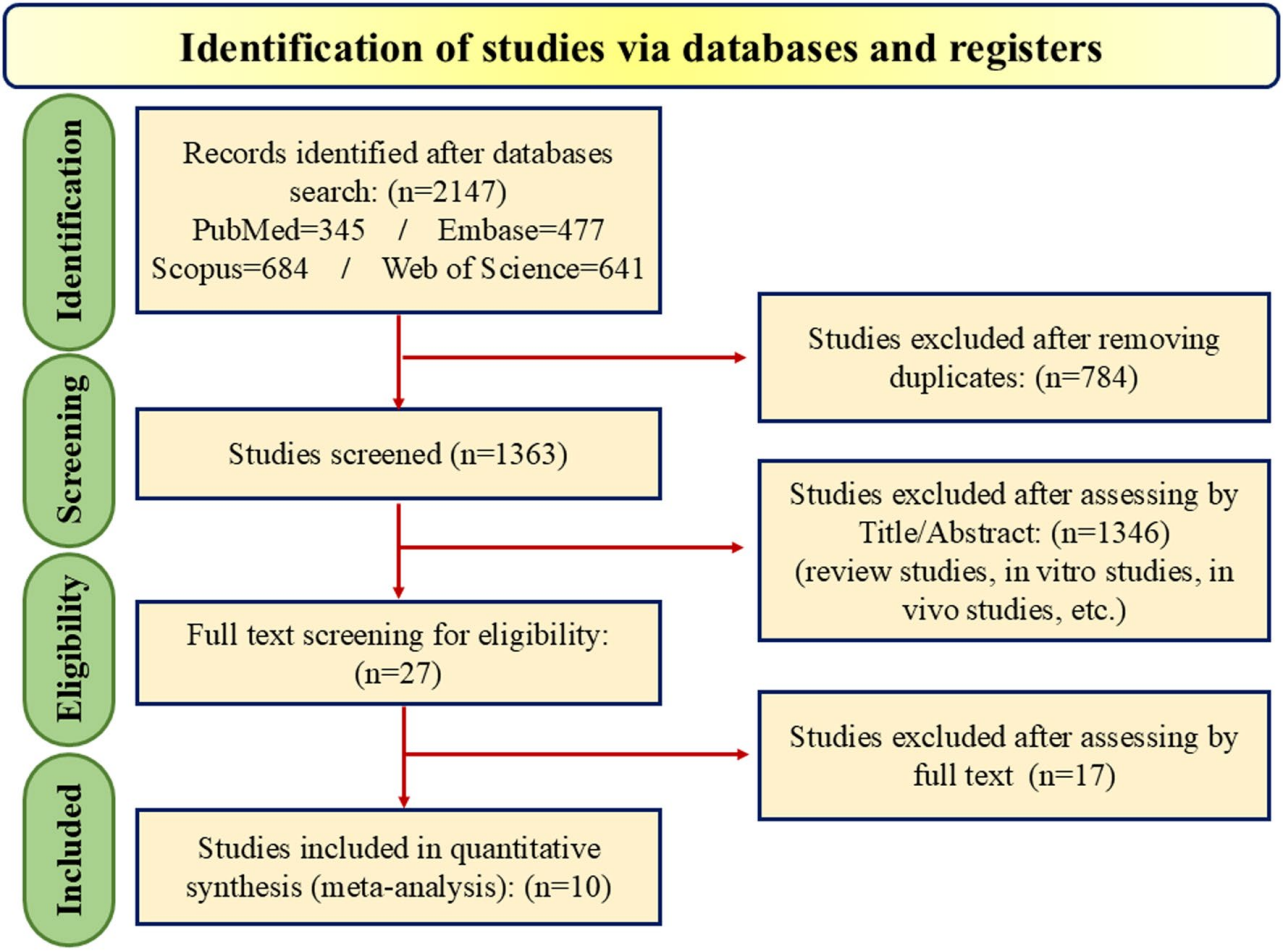
Flowchart of study selection for including trials in the systematic review

A comprehensive search of four databases, including PubMed, Web-of-Science, Scopus, Embase, and a manual search in Google Scholar, yielded a total of 2147 articles. After the removal of 784 duplicate records, 1363 unique studies were subjected to the selection process. The initial screening of titles and abstracts excluded 1346 papers from 1363 studies because of exclusion criteria (review studies, non-human studies, not being RCT, etc.), and 27 studies had inclusion criteria. This left 27 papers for full-text assessment, during which 17 more publications were excluded due to reasons such as Not being RCT (n = 5), inadequate data (n = 4), not being overweight (n = 5), or lack of relevance to the research question (n = 3). Consequently, ten studies were deemed suitable for data extraction and included all RCTs in the final quantitative meta-analysis (Fig. 2). These 10 RCTs formed the basis for the meta-analysis, providing robust data for evaluating the research hypotheses.

### Study characteristics

The review included studies published from 2007 to 2023, encompassing 424 participants: 210 in intervention and 214 in control groups. Study durations ranged from 10 to 24 weeks, and participants' ages varied from 8 to 64 years.

Most studies assessed chia seed consumption in grams per day (gr/day). Chia seeds were supplied in several forms, including whole, ground, and chia seed oil. The outcome measurements were cardiometabolic indices and risk factors for metabolic and cardiovascular diseases, including anthropometric index (body weight, BMI, WC), BP, lipid profile (TG, TC, HDL, LDL), glycemic markers (FBG, Hb1AC, Insulin), and inflammatory markers (CRP, IL-6, TNF-α) (Table 2).

**Table 2:** General characteristics of included studies in the meta-analysis

Author/year/Ref	Country	Study design	Population	Sample size (Int./Cont.)	Age (Int./Cont.)	Gender (male/female)	Male	Type of Chia	Dose (gr/day)	Duration
Vuksan et al 2007 [42]	Canada	RCT-SB Cross-over	Overweight T2DM	20 (Int.:10/Cont.: 10)	Int.: 64 (± 8) Cont.: 64 (± 8)	11/9	55%	Chia seeds	37	24 weeks
Nieman et al (male) 2009 [30]	USA	RCT-SB	Overweight Obese	28 (Int.:14/Cont.: 14)	Range: 20–70	28/0	100%	Chia seeds	50	12 weeks
Nieman et al (female) 2009 [30]	USA	RCT-SB	Overweight Obese	48 (Int.:25/Cont.: 23)	Range: 20–70	0/48	0%	Chia seeds	50	12 weeks
Nieman et al 2012 [38]	USA	RCT-DB	Overweight Obese Postmenopausal	42 (Int.:16/Cont.: 26)	Int.: 60.4 (± 6.4) Cont.: 58.5 (± 5.6)	0/56	0%	Whole Chia seeds	25	10 weeks
Nieman et al 2012 [38]	USA	RCT-DB	Overweight Obese Postmenopausal	40 (Int.:14/Cont.: 26)	Int.: 57.2 (± 6.36) Cont.: 58.5 (± 5.6)	0/56	0%	Miled Chia seeds	25	10 weeks
Brissette et al 2013 [64]	Canada	RCT-DB	Overweight Obese T2DM	58 (Int.:27/Cont.: 31)	Int.: 60 (± 1.6) Cont.: 60 (± 1.6)	18/40	31%	Chia seeds	30	24 weeks
Toscano et al. 2014 [39]	Brazil	RCT-DB	Overweight Hypertension	26 (Int.:19/Cont.: 7)	Int.: 48.8 (± 7.84) Cont.: 51.4 (± 8.2)	NR	NR	Chia flour	35	12 weeks
Toscano et al. 2015 [31]	Brazil	RCT-DB	Overweight Obese	26 (Int.:19/Cont.: 7)	Int.: 48.8 (± 8.71) Cont.: 51.4 (± 13.07)	NR	NR	Chia flour	35	12 weeks
Vuksan et al. 2017 [16]	Canada	RCT-DB	Overweight Obese T2DM	58 (Int.27/Cont.: 31)	Int.: 60 (± 2) Cont.: 60 (± 2)	18/40	31%	Chia seeds	30	24 weeks
C. S. da Silva et al. 2020 [65]	Brazil	RCT-DB	Overweight Obese children	16 (Int.:8/Cont.: 8)	Int.: 8.62 (± 1.69) Cont.: 8.75 (± 1.59)	8/8	50%	Chia seeds	25	11 weeks
Alwosais et al 2021 [66]	Kuwait	RCT-DB	Overweight T2DM	42 (Int.:20/Cont.: 22)	Int.: 51.8 (± 8.9) Cont.: 52.7 (± 7.3)	22/20	52%	Chia seeds	40	12 weeks
Quaresma et al. 2023 [67]	Brazil	RCT-SB	Overweight Obese	20 (Int.:11/Cont.: 9)	NR	0/20	0%	Chia flour	30	12 weeks

Ref.: references; RCT: randomized clinical trial; SB: Single-blind; DB: Double-blind; Int.: Intervention; Cont.: Control; T2DM: type 2 diabetes mellitus; NR: not reported; (mean, SD)

Vuksan, V., et al., [42], Nieman, D.C., et al., [30], Nieman, D.C., et al., [38], Brissette, C., [64], Toscano, L.T., et al. [39], Toscano, L.T., et al., [31], Vuksan, V., et al., [16], da Silva, C.S., et al., [65], Alwosais, E.Z.M., et al., [66], Quaresma, L.S., et al., [67]

### Effect on anthropometric indices (BMI and WC)

In this meta-analysis, five and four studies examined the effect of chia supplementation on BMI and WC, respectively. They concluded that there was no statistically significant (MD = 0.1 kg/m^2^, 95% CI [− 0.36, 0.43], I^2^ = 12.58%, p = 0.86) effect on BMI compared to the control group (Fig. 2A). However, the intervention group experienced a significant − 2.82 cm decrease in WC compared to the control group (MD = − 2.82 cm, 95% CI [− 4.32, − 1.31], I^2^ = 0.0%, p < 0.001) (Fig. 3B).

**Fig . 3:**
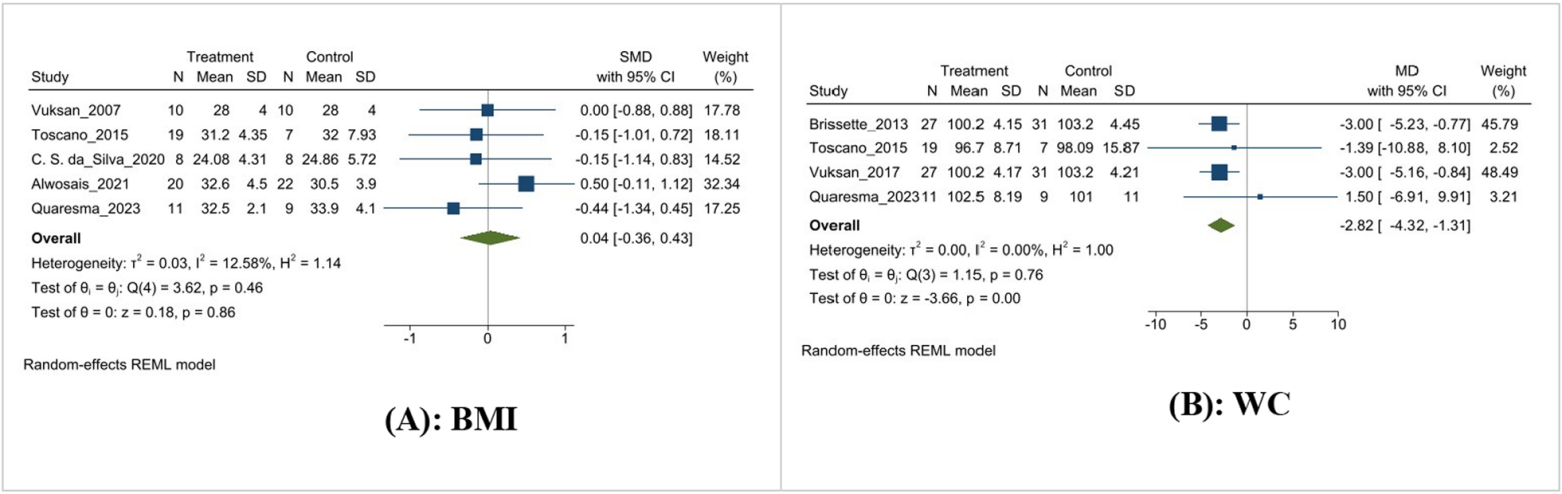
Effect of Chia supplementation on anthropometric indices; **A**: BMI; **B**: WC

### Effect on blood pressure (BP)

**Fig. 4:**
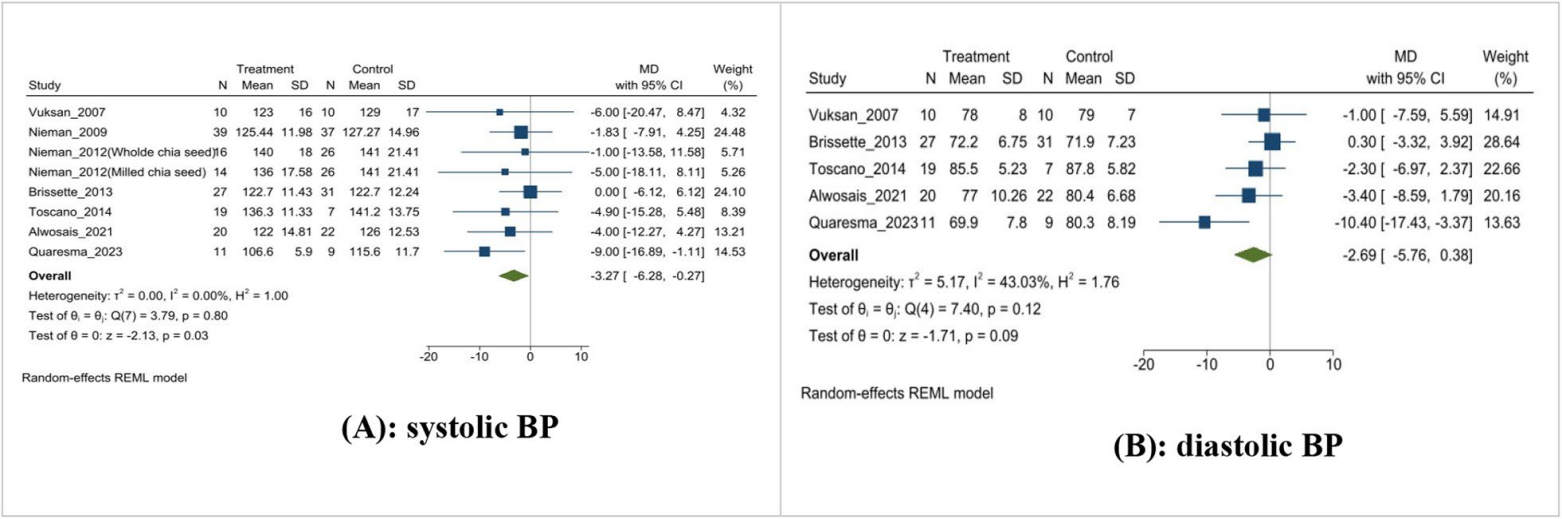
Effects on blood pressure. **A**: systolic BP; **B**: diastolic BP

In this meta-analysis, seven studies examined the effect of chia seed supplementation on systolic BP, while five examined its impact on diastolic BP. The intervention group experienced a significant (MD: − 3.27 mmHg, 95% CI [− 6.28, − 0.27], I^2^ = 0.0%, p-value = 0.03) reduction of − 3.27 mmHg in systolic BP compared to the control group (Fig. 4A). On the other hand, the intervention group showed a slight non-significant (MD: − 2.69 mmHg, 95% CI [− 5.76, 0.38], I^2^ = 43.03%, p = 0.09) reduction in diastolic BP compared to the control group (Fig. 4B).

### Effect on lipid profile

**Fig. 5:**
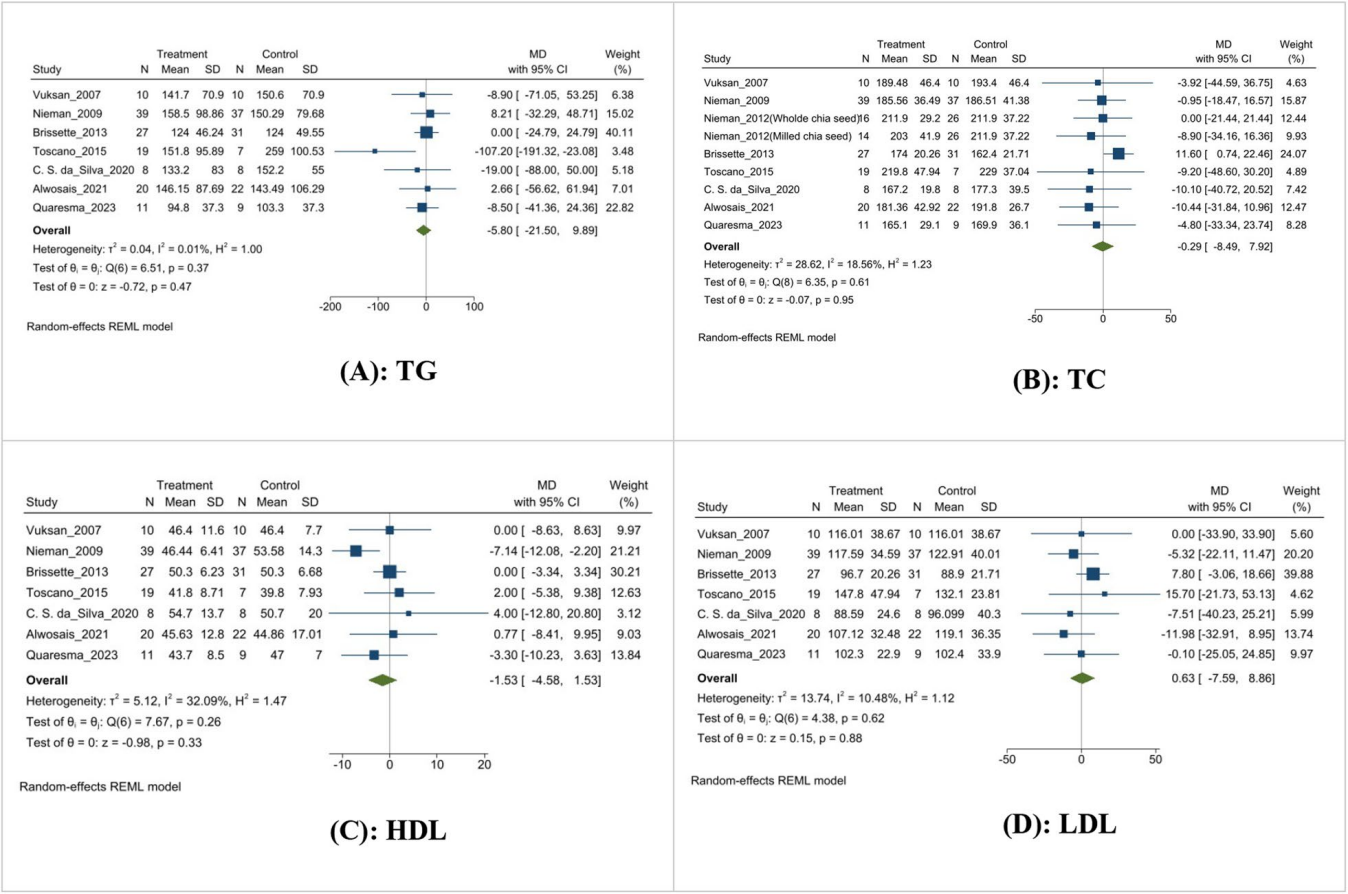
Effect of chia supplementation on lipid profile. **A**: TG; **B**: TC; **C**: HDL; **D**: LDL

In this meta-analysis, seven studies reported triglycerides (TG), in which 5.80 mg/dL was reduced, but it was not significant (MD: − 5.80 mg/dL, 95% CI [− 21.50, 9.89], I^2^ = 00.01%, p = 0.47) (Fig. 5A). Similarly, eight studies reported that total cholesterol (TC) 5.80 mg/dL was reduced, but it was not significant (MD: − 0.29 mg/dL, 95% CI [− 8.49, 7.92], I^2^ = 18.56%, p = 0.95) (Fig. 5B). Seven studies reported mean differences in high-density lipoprotein (HDL) and low-density lipoprotein (LDL). HDL had a non-significant decrease of 1.53 mg/dL (MD: − 1.53 mg/dL, 95% CI [− 4.58, 1.53], I^2^ = 32.09%, p = 0.33) (Fig. 5C), and LDL exhibited a non-significant increase of 0.63 mg/dL (MD: 0.63 mg/dL, 95% CI [− 7.59, 8.86], I^2^ = 10.48%, p = 0.88) (Fig. 5D).

### Effect on glycemic markers

**Fig. 6:**
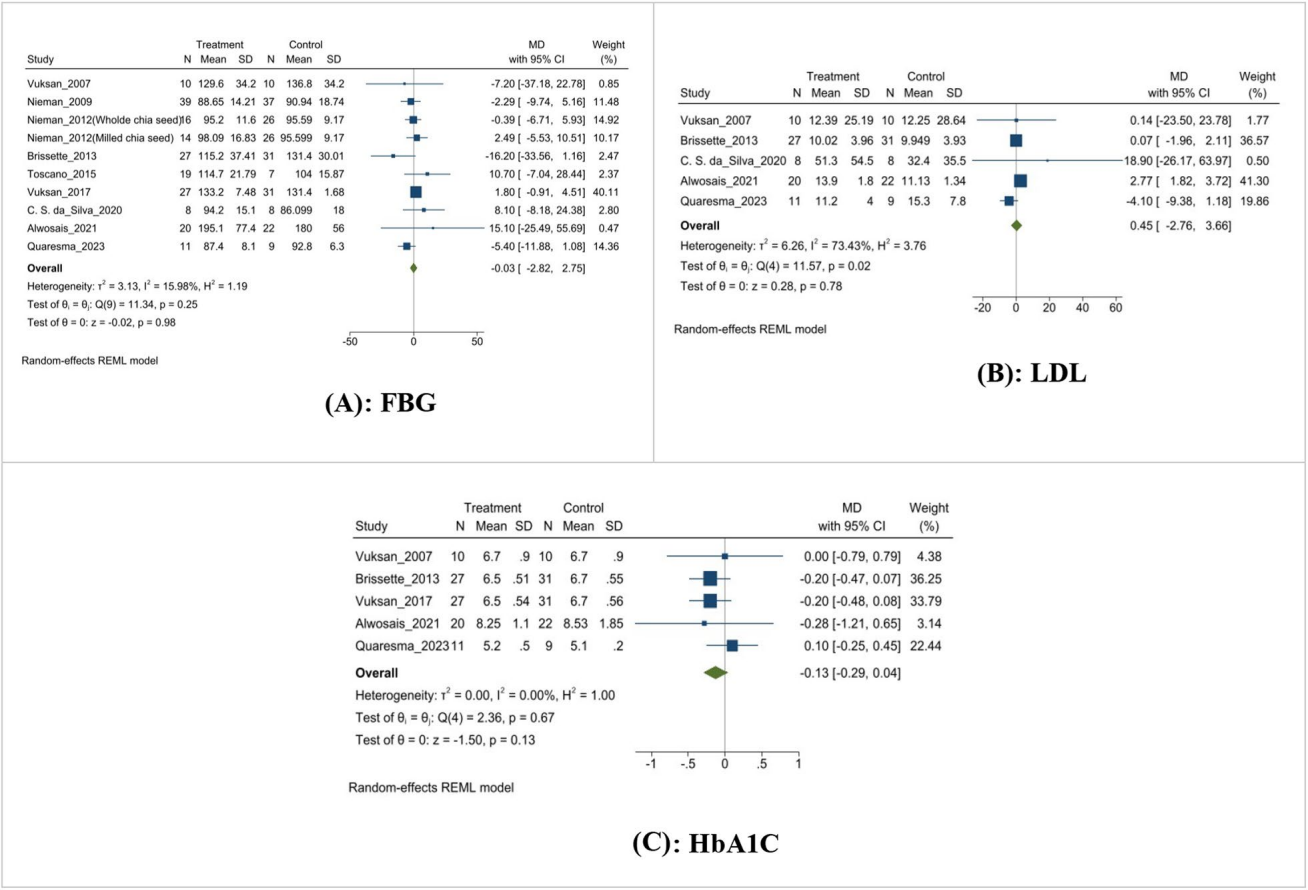
Effect of chia supplementation on glycemic markers. **A**: FBG; **B**: Insulin; **C**: Hb1AC

The meta-analysis examining the effects of chia seed supplementation in overweight subjects reveals no significant impact on fasting blood glucose (FBG), hemoglobin A1c (HbA1c), or insulin levels. Specifically, FBG showed a negligible with a mean difference of − 0.03 mg/dL (MD: − 0.03 mg/dL, 95% CI [− 2.82, 2.75], I^2^ = 15.98%, p = 0.98) (Fig. 6A), insulin levels exhibited a non-significant mean difference of 0.45 µIU/mL (MD: 0.45 µIU/mL, 95% CI [− 2.76, 3.66], I^2^ = 73.43%, p = 0.78) (Fig. 6B), and HbA1c had a non-significant mean reduction of − 0.13% (MD: − 0.13%, 95% CI [− 0.29, 0.04], I^2^ = 0.0%, p = 0.13) (Fig. 6C). These results suggest that chia seed supplementation does not significantly alter these glycemic and insulin parameters in overweight individuals (Fig. 6).

### Effect on Inflammatory markers

**Fig. 7:**
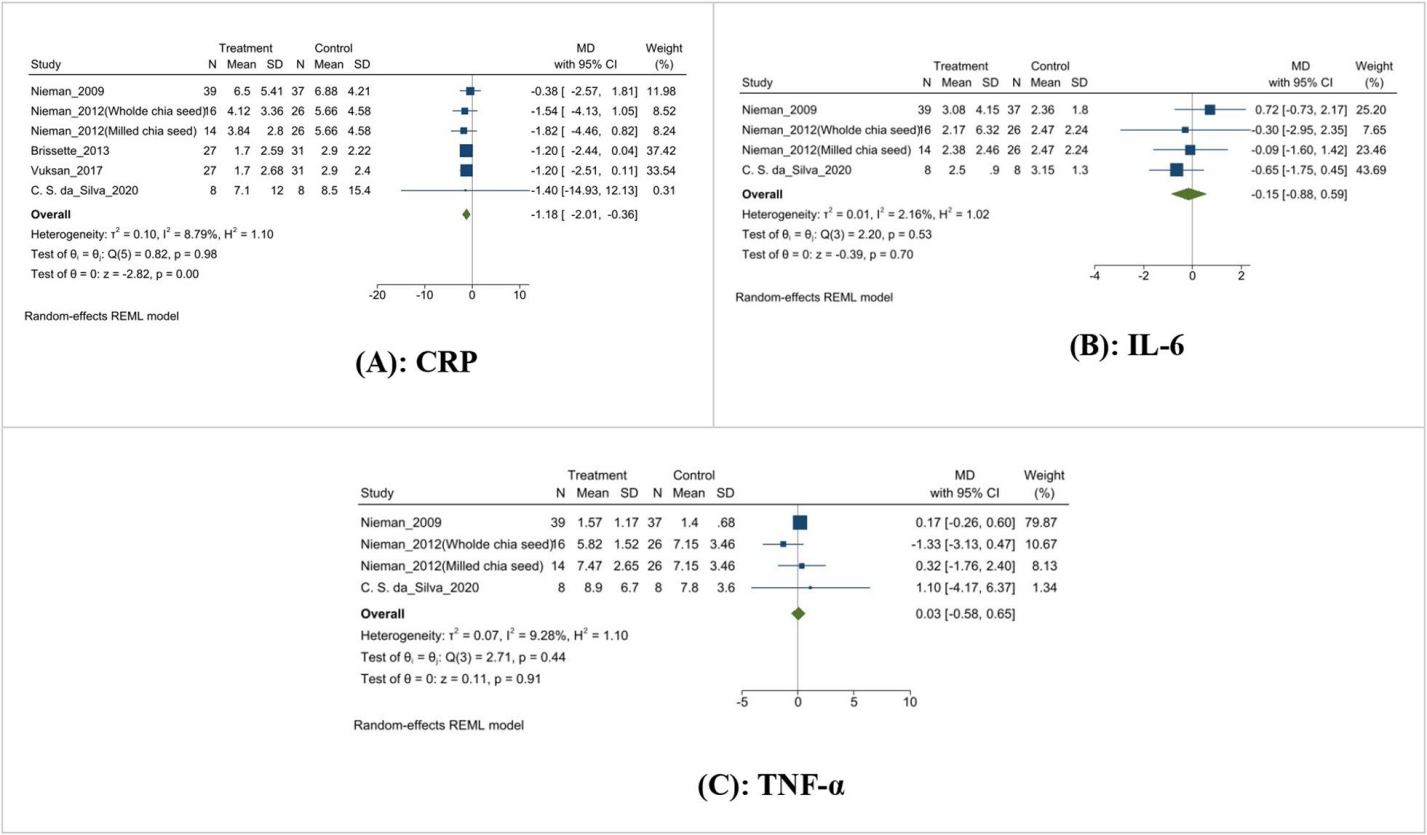
Effect of chia supplementation on Inflammatory markers. **A**: CRP; **B**: IL-6; **C**: TNF-α

The meta-analysis on chia seed supplementation in overweight subjects shows a significant reduction in C-reactive protein (CRP) levels, with a mean difference of − 1.18 mg/L (MD: − 1.18 mg/L, 95% CI [− 2.01 to − 0.36], I^2^ = 87.9%, p < 0.0001) (Fig. 7A), indicating a substantial effect on reducing inflammation. However, there were no significant changes in interleukin-6 (IL-6) (MD: − 0.15 pg/ml, 95% CI [− 0.88 to 0.59], I^2^ = 2.16%, p = 0.7) (Fig. 7B), and tumor necrosis factor-alpha (TNF-α) levels (MD: 0.03 pg/ml, 95% CI [− 0.58 to 0.65], I^2^ = 9.28%, p = 0.91) (Fig. 7C), suggesting that chia seed supplementation does not significantly impact these inflammatory markers.

### Heterogeneity and sensitivity analysis

The heterogeneity among studies varied in outcome measures, but it was generally low to moderate (I^2^ < 50%). Despite excluding studies with a high risk of bias, the overall effect sizes were equivalent, and sensitivity analyses assessed the robustness of the results. The sensitivity analysis showed that excluding any individual study did not impact the effect size of chia seed supplementation in outcomes such as FBG.

### Publication bias

**Fig. 8:**
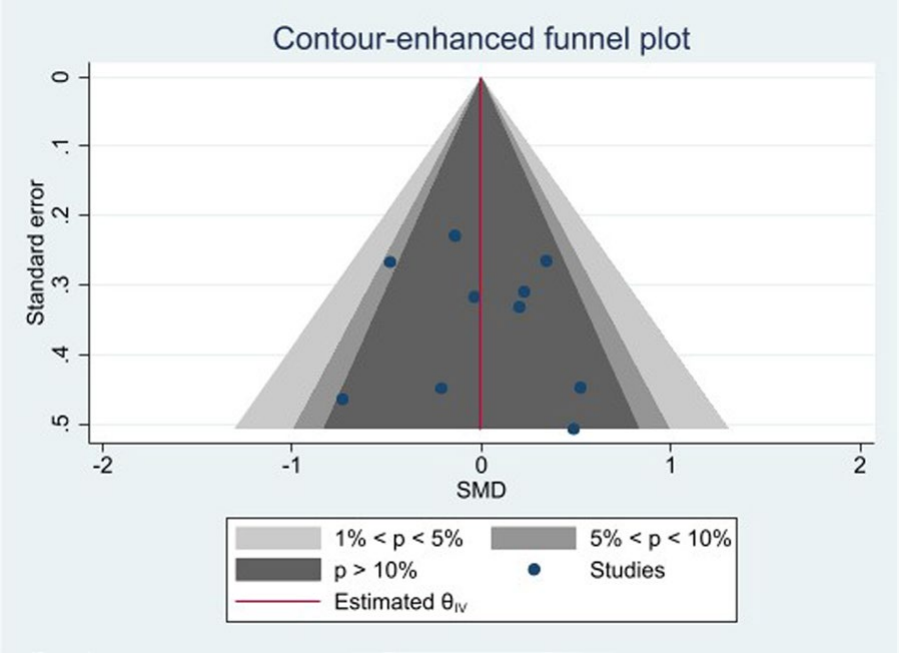
Funnel plot for FBG

A funnel plot was designed to assess the parameter FBG, which was evaluated in at least ten studies. Figure 8 illustrates a moderate degree of asymmetry, indicating the presence of publication bias, possibly resulting from the difficulties in disseminating negative or inconclusive findings. Based on most outcome measures, the examination of funnel plot asymmetry and Egger's regression test implementation failed to find any significant indications of publication bias in the included papers. However, there was a notable publication bias for FBG, as indicated by a t-value of 3.13 and a P-value of 0.98.

### Subgroup analysis

**Fig. 9:**
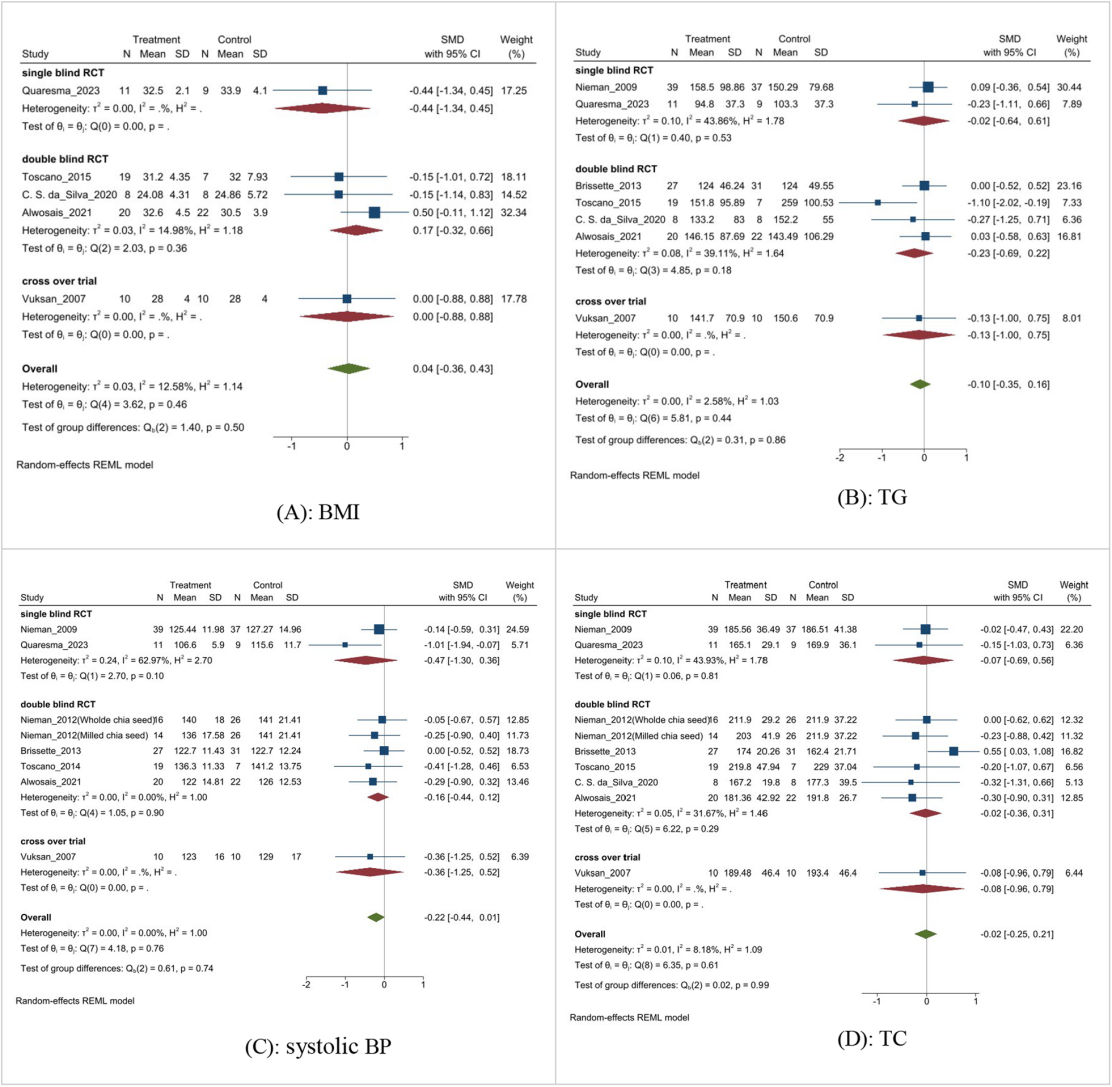

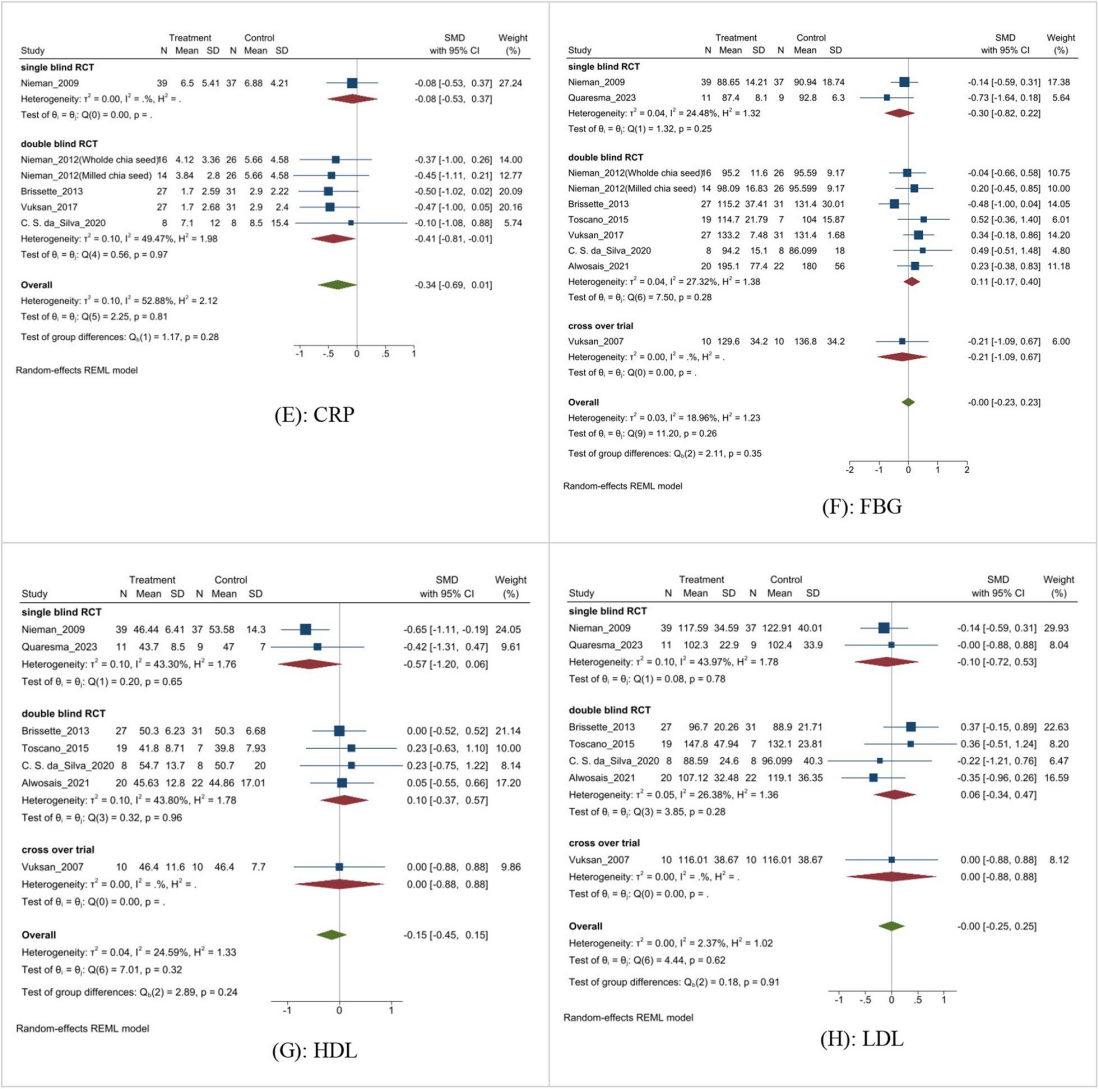
Subgroup analysis.** A**: BMI; **B**: TG; **C**: systolic BP; **D**: TC;** E**: CRP;** F**: FBG; **G**: HDL; **H**: LDL

Subgroup analyses were performed to determine the source of high heterogeneity in the meta-analysis. The results of the subgroup analysis based on the BMI are shown in Fig. 9A. Subgroup analysis indicated that there were no statistically significant differences among different study designs, such as single-blind RCTs, double-blind RCTs, and cross-over trials (MD: 0.04, 95% CI − 0.36 to 0.43, p = 0.46, I^2^ = 12.58%). Similarly, subgroup analysis for TG (Fig. 9B) and systolic BP (Fig. 9C) revealed no significant differences among different studies (MD: − 0.10, 95% CI − 0.35 to 0.16, p = 0.44, I^2^ = 2.58%), (MD: − 0.22, 95% CI − 0.44 to 0.01, p = 0.76, I^2^ = 0%), respectively.

Stratifying the analysis based on study designs revealed no difference in TC (Fig. 9D) among different studies (MD: − 0.02, 95% CI − 0.25 to 0.21, p = 0.61, I^2^ = 8.81%). Subgroup analysis showed a negligible difference in CRP (Fig. 9E) among different study designs (MD: − 0.34, 95% CI − 0.69 to 0.01, p = 0.81). Based on the study designs, the subgroup analysis showed no significant difference in FBG (Fig. 9F) among studies (MD: − 0.0, 95% CI − 0.23 to 0.23, p = 0.26).

When analyzing the lipid profile, subgroup analysis did not reveal significant differences in LDL (Fig. 9G) or HDL (Fig. 9H) levels across various trial designs. The subgroup analysis found no statistically significant difference in different study designs (mean difference: − 0.22, 95% CI − 0.44 to 0.01, p = 0.76).

## Discussion

This systematic review and meta-analysis included ten RCTs with 424 participants to assess the effects of chia seed supplementation on cardiometabolic indices in overweight subjects. The meta-analysis findings indicate that chia seed supplementation does not significantly change lipid profile parameters such as TG, TC, HDL, or LDL, nor does it change glycemic markers, including FBG, HbA1c, or insulin levels. However, chia seeds are associated with a significant reduction in CRP, an inflammatory marker, and a decrease in WC and systolic BP. Despite these positive effects, no significant changes were observed in IL-6, TNF-α, BMI, or diastolic BP. The included studies exhibited low to moderate heterogeneity in outcome measures.

Our study indicates that consuming 30 g/day of chia seeds for six months may reduce weight and WC in overweight individuals. Some previous studies support our findings, while others do not [27, 28]. For example, Guevara-Cruz et al. [29] demonstrated a significant decrease in WC in individuals with metabolic syndrome who consumed a regular diet, including chia seeds, soy, nopal, and oats. Conversely, Nieman et al. [30] found that consuming 50 g/day of chia for 12 weeks did not result in weight loss among obese or overweight individuals. Toscano et al. [31] reported a notable decrease in WC among overweight and obese individuals who consumed 35 g/day of chia for twelve weeks. However, another study found no significant changes in weight, BMI, WC, or insulin resistance after a 10-day consumption of 25 g/day of chia [32]. Lovato's research revealed significant reductions in body weight, abdominal circumference, WC, and BMI after chia intervention [33]. Similarly, Ayaz et al. [34] showed significant weight, BMI, and WC decreases after a six-month diet, including 25 g/day of chia. The differing outcomes across studies could be attributed to duration, dosage, and variations in participants’ characteristics. While chia seeds are high in dietary fiber, which can promote fullness and potentially aid in weight reduction, several factors contribute to this. Factors such as participant characteristics (like baseline metabolic rate and lifestyle habits), adherence to the supplementation regimen, and variations in measurement and reporting methods can all influence the effectiveness of chia seeds in achieving weight reduction [34–37].

Previous studies have highlighted the beneficial components of chia seeds for cardiovascular health, including their high insoluble fiber content, which more effectively lowers BP than soluble fiber [29, 38–41]. Vuksan et al. [42] found that chia seed consumption reduced systolic BP in individuals with well-managed T2DM. However, other studies reported no significant changes in systolic BP after 10 or 12 weeks of chia supplementation [30, 31].

Chia seeds have several compounds that positively impact BP and cardiovascular health. They are rich in crucial fatty acids, particularly with a high omega-3 to omega-6 ratio, linked to reduced BP and a lower risk of CVD [43–45]. A 100-g serving of chia provides significant amounts of magnesium, potassium, and calcium, all potent vasodilators and vascular smooth muscle contraction inhibitors, effectively reducing BP [46–49]. Additionally, chia seeds contain flavonoids such as quercetin, chlorogenic acid, and caffeic acid, which have been shown to lower BP [14, 43]. Research by Toscano et al. [39] demonstrated that chia flour intake consistently reduced BP in hypertensive individuals. The dietary fiber in chia seeds forms a gel-like substance when combined with water, aiding in satiety, reducing hunger, and potentially lowering the risk of coronary heart disease. This fiber also acts as a prebiotic, beneficial for treating hypertension and obesity [14, 43].

The chia seeds help slow down starch digestion and reduce post-meal blood glucose levels [8, 50]. Chia seeds include viscous and soluble fibers that have been shown to reduce cholesterol levels by affecting the metabolism of lipids in the liver [51]. This impact is accomplished by increased excretion of bile acids, reducing the uptake of cholesterol and other lipids in circulation, and inhibiting the production of free fatty acids in the liver [43]. Furthermore, the functional proteins and bioactive peptides found in chia can block the activity of 3 hydroxy 3 methylglutaryl coenzyme reductase, which plays a crucial role in forming cholesterol. As a result, the synthesis of cholesterol is diminished [52].

Sosa-Crespo et al. [53] demonstrated that consuming chia seeds within 30–60 min lowers plasma glucose levels in healthy individuals and those with prediabetes or T2DM. Vuksan et al. [17] also found that various doses of chia (7, 15, and 24 g) reduced postprandial blood glucose levels in healthy individuals. Additionally, a previous meta-analysis reported that higher doses of chia seeds reduced postprandial glucose levels [9]. FBG reductions were observed in several subgroups, including healthy women, overweight or obese individuals, hypertensive patients, and those with metabolic syndrome [29, 38, 39]. However, in our study, chia seed supplementation showed a slight improvement in glycemic markers among overweight individuals, but this improvement was not statistically significant. Several factors may account for this discrepancy. First, the inclusion of only 10 RCTs may have been insufficient to detect significant changes in glycemic markers. Second, differences in baseline characteristics of our study population compared to those in other studies could have influenced the outcomes. Third, variations in the amount and form of chia seed consumption and unaccounted dietary and lifestyle factors may have affected the results. Finally, individual variability in response to chia seed consumption could also explain our study's lack of significant findings.

The bioactive components in chia seeds have been linked to preventive effects against obesity and plasma oxidative stress in several studies [12, 54]. Obesity is characterized by persistent, low-level inflammation in adipose tissue, accompanied by the excessive production of pro-inflammatory adipokines [55, 56]. Elevated levels of these inflammatory mediators in the bloodstream have been linked to the activation of hepatocytes, prompting them to create CRP [54]. Elevated CRP levels are associated with a higher risk of developing coronary heart disease and increased mortality rates [57].

Although the exact anti-inflammatory mechanism of chia seeds is not fully understood, the high levels of alpha-linolenic acid (ALA) in chia seeds may suggest an anti-inflammatory effect [58]. However, the primary method of enhancing antioxidant levels in plasma is still debated, as it is hypothesized that there may be a decrease in pro-oxidative substances or an increase in antioxidant substances [59]. The antioxidant capacity of chia seeds is defined by their ability to inhibit the synthesis of reactive oxygen species (ROS) or through direct scavenging action. These antioxidants can transform into active molecules, reducing cell damage and playing a role in regulating gene expression and signal transduction. Consequently, the body initiates feedback processes to enhance cellular defense and survival [60]. A study showed that chia's anti-inflammatory effect seems to be associated with the inhibition of PPARγ and NF-κB expression (61). Furthermore, research has shown that chia seeds contain many phenolic compounds and high levels of natural antioxidants, including quercetin and kaempferol, while caffeic and chlorogenic acids are in minimal quantities [62]. These antioxidative properties make chia seeds a valuable source of antioxidants [63].

This meta-analysis used data only from RCTs, which are considered the most rigorous form of clinical scientific evidence. Our investigation did not uncover any indications of publication bias that could have affected the meta-analysis's conclusions. We also tried to minimize any potential bias in the review process by conducting an in-depth assessment of existing literature and adhering to the rigorous PRISMA requirements for conducting and reporting the review. In addition, we performed subgroup analyses to examine the impacts within distinct subgroups and identify the probable variables that may contribute to the found heterogeneity. These methodological strengths enhance the reliability of our findings, providing robust evidence to support clinical decision-making.

Despite these strengths, our study has several limitations that warrant careful consideration. The overall sample size was small, potentially affecting the statistical power of our results. Additionally, significant heterogeneity was observed, likely due to variations in study populations, dosing regimens, and treatment durations, which complicates the interpretation of the findings. Most of the included studies originated from Eastern countries, raising concerns about the generalizability of our results to Western populations. Furthermore, several outcomes were assessed by only a few studies, limiting the strength of the evidence supporting those conclusions. These factors may undermine the validity of our results and should be acknowledged when interpreting our findings.

## Conclusions

The findings of this meta-analysis indicate that chia seed supplementation does not lead to significant changes in most lipid profile parameters, including TG, TC, HDL, and LDL, nor in FBG, HbA1c, or insulin levels. Despite these findings, chia seeds appear to have beneficial effects on certain health markers. Specifically, chia seed supplementation was associated with a notable reduction in CRP, suggesting a potential anti-inflammatory effect. Additionally, significant reductions in waist circumference and systolic blood pressure were observed, though changes in diastolic blood pressure were not significant. No substantial impact on BMI was found. Overall, chia seeds should be considered a valuable adjunct in managing cardiometabolic health, particularly due to their anti-inflammatory properties and effects on WC and PB. However, they should not be relied upon as a sole intervention. Chia seeds should be integrated into a comprehensive health strategy that includes a balanced diet, regular physical activity, and other lifestyle modifications for optimal health outcomes.

Future studies should aim to include larger RCTs and more diverse sample sizes with standardized dosing regimens and treatment durations to enhance statistical power, reduce heterogeneity, and improve the generalizability and validity of the findings.

## Supplementary Information

Additional file1

## Data Availability

The study includes the original contributions in the article/Supplementary material. For further inquiries, please contact the corresponding authors.

## Abbreviations

CVD: Cardiovascular diseases
BMI: Body Mass Index
WC: Waist circumference
mm Hg: Millimeters of mercury
BP: Blood pressure
FBG: Fasting blood glucose
Hb1AC: Hemoglobin A1c
TG: Triglycerides
TC: Total cholesterol
HDL: High-density lipoprotein
LDL: Low-density lipoprotein
CRP: C-reactive protein
IL-6: Interleukin-6
