# Supplementary material for: Effects of chia seed (Salvia hispanica L.) supplementation on cardiometabolic health in overweight subjects: a systematic review and meta-analysis of RCTs
Source: Nutr Metab (Lond). 2024 Sep 16;21:74. doi: 10.1186/s12986-024-00847-3 (PMC11406937; doi:10.1186/s12986-024-00847-3)
Supplement: Supplementary file 1 — Additional file1 [file 12986_2024_847_MOESM1_ESM.docx]

**Supplementary Materials**

**Query words:**

**Chia:**

“chia” OR “salba” OR “salvia” OR “salba-chia”

**Cardiometabolic:**

“cardiometabolic” OR “metabolic” OR “metabolism” OR “Lipid” OR “Lipemia*” OR “Lipidemia*” OR “Hyperlipemia*” OR “Hyperlipidemia” OR “Hypolipemia” OR “Hypolipidemia” OR “Cholesterol” OR “Triglyceride*” OR “Triacylglycerol*” OR “lipoprotein*” OR “HDL” OR “High-density Lipoprotein” OR “LDL” OR “low-density lipoprotein” OR “Hypercholesteremia*” OR “Dyslipidem*” OR “Dyslipoproteinemia*” OR “hyperlipoproteinemia” OR “hyperlipidemic agents” OR “Apolipoprotein*” “alpha Lipoprotein” OR “Circulating Lipoprotein” OR “Lipid metabolism disorder” OR “Glycemic” OR “glycemia” OR “glucose” OR “blood sugar” OR “FBS” OR “FBG” OR “HbA1C” OR “hemoglobin A1C” OR “Glycohemoglobin*” OR “Glycated hemoglobin*” OR “Glycosylated Hemoglobin A1c” OR “Hyperglycemia” OR “Hypoglycemia” OR “inflammatory marker” OR “C-reactive protein” OR “CRP”

**Table.** Ris of Bias (RoB2)

| **Ris of Bias (RoB2)** | | | | | | |
| --- | --- | --- | --- | --- | --- | --- |
| **Author/Year** | **D1** | **D2** | **D3** | **D4** | **D5** | **Overall** |
| Quaresma et al. 2023 | L | Some concerns | L | L | Some concerns | Some concerns |
| Alwosais et al. 2021 | L | L | L | L | Some concerns | Some concerns |
| C. S.da Silva et al. 2020 | L | L | L | L | Some concerns | Some concerns |
| Vuksan et al. 2017 | L | L | L | L | Some concerns | Some concerns |
| Toscano et al. 2015 | L | L | L | L | Some concerns | Some concerns |
| Toscano et al. 2014 | L | L | L | L | Some concerns | Some concerns |
| Brissette et al. 2013 | L | L | L | L | L | L |
| Nieman et al. 2012 | L | L | L | L | Some concerns | Some concerns |
| Nieman et al. 2009 | L | L | L | L | Some concerns | Some concerns |
| Vuksan et al. 2007 | L | Some concerns | L | L | Some concerns | Some concerns |

L: low risk


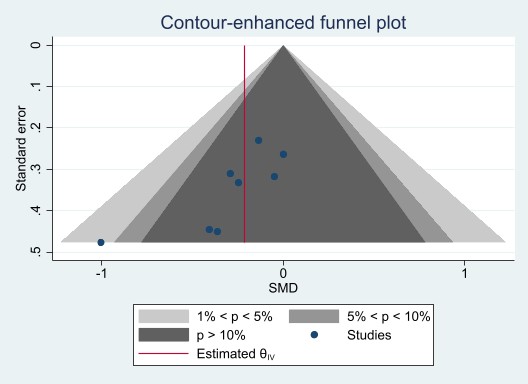


**Figure.** Funnel plot for SBP

**Sensitive analysis**

**
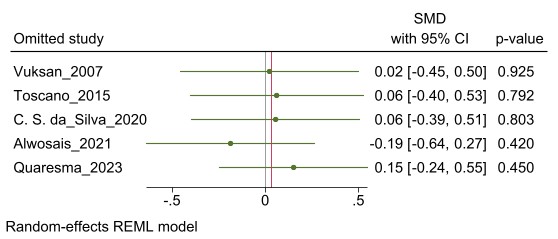
**

**Sensitivity analysis of BMI**

**
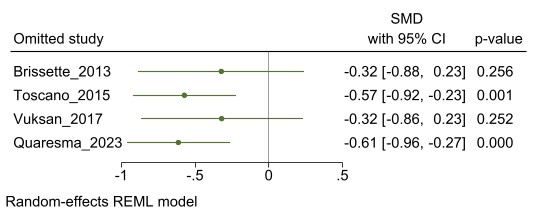
**

**Sensitivity analysis of WC**

**
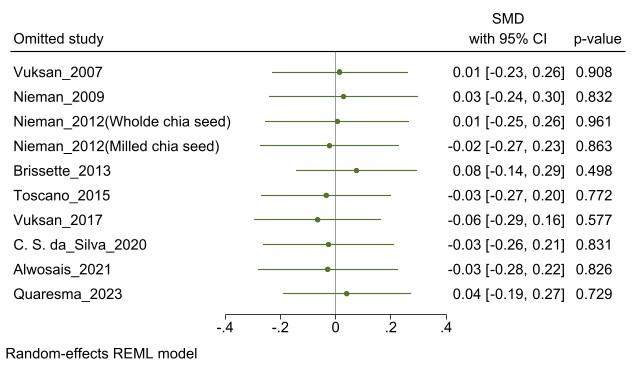
**

**Sensitivity analysis of FBG**

**
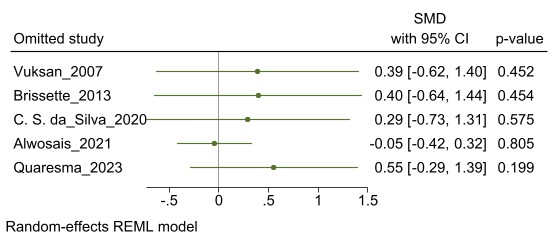
**

**Sensitivity analysis of insulin**

**
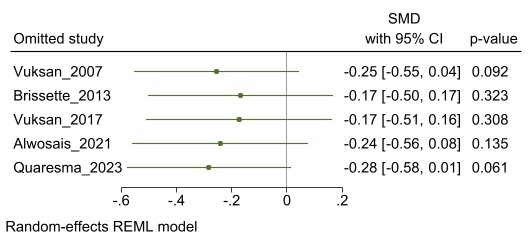
**

**Sensitivity analysis of HbA1C**

**
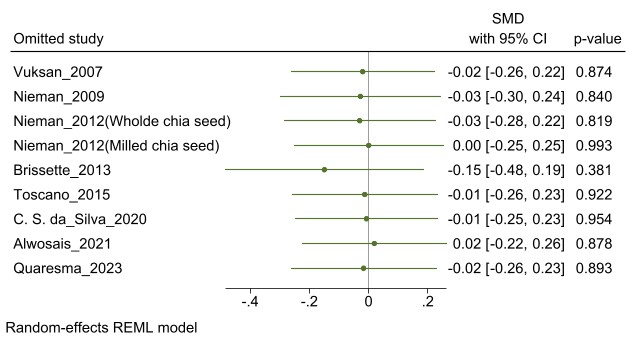
**

**Sensitivity analysis of TC**

**
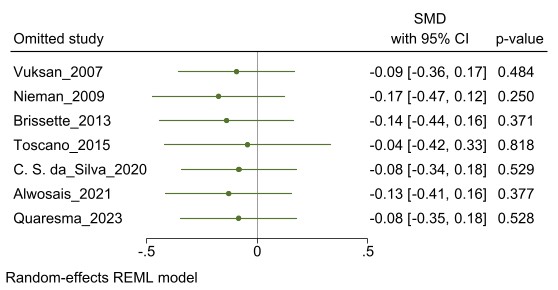
**

**Sensitivity analysis of TG**

**
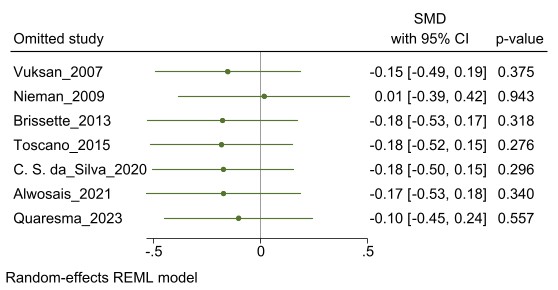
**

**Sensitivity analysis of HDL**

**
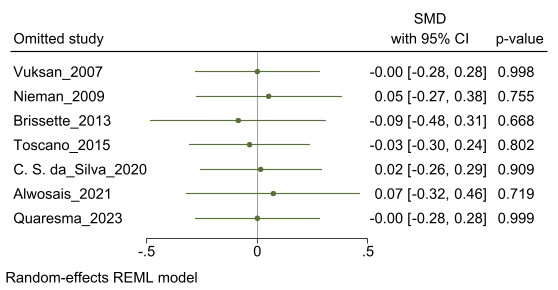
**

**Sensitivity analysis of LDL**

**
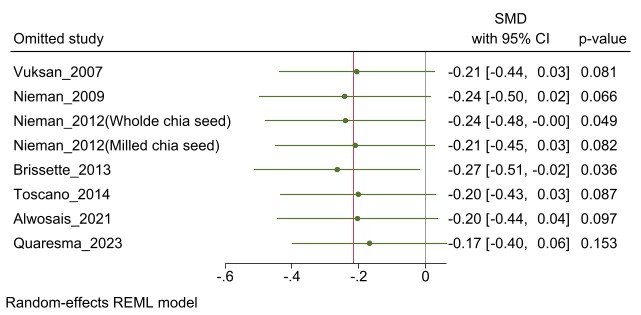
**

**Sensitivity analysis of systolic BP**

**
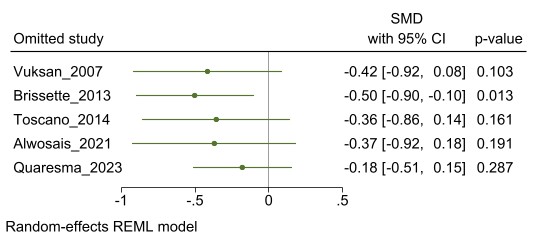
**

**Sensitivity analysis of diastolic BP**

**
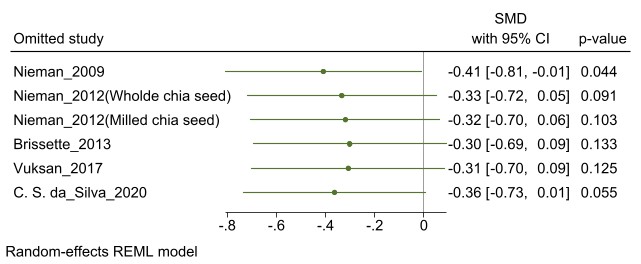
**

**Sensitivity analysis of CRP**

**
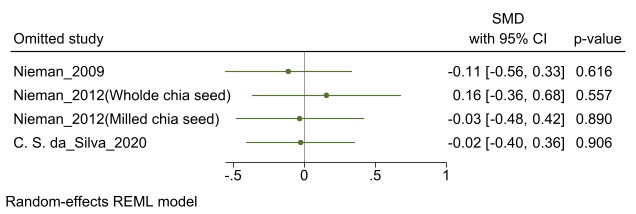
**

**Sensitivity analysis of TNF-a**

**
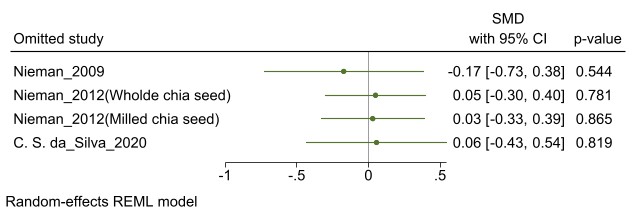
**

**Sensitivity analysis of IL-6**

**Egger Regression test**

**
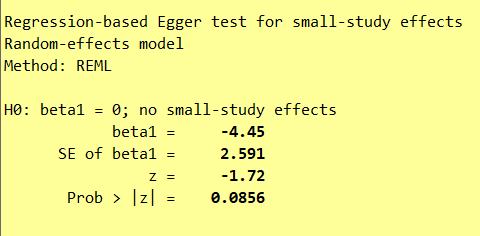
**

**
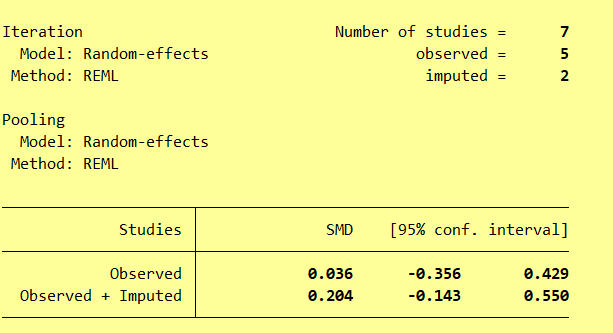
**

**Egger Regression test of BMI**

**
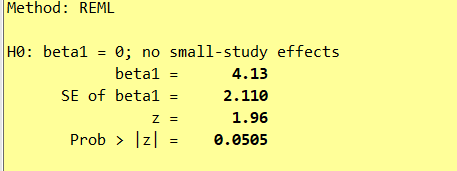
**

**
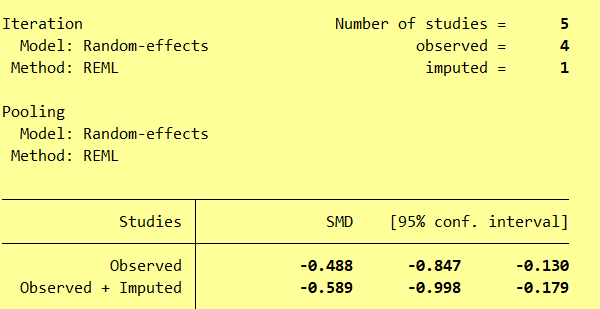
**

**Egger Regression test of WC**

**
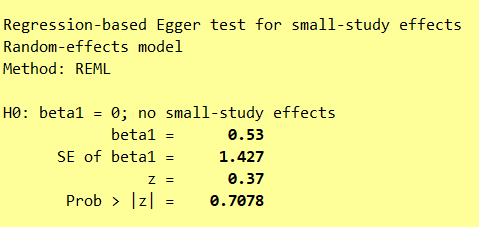
**

**
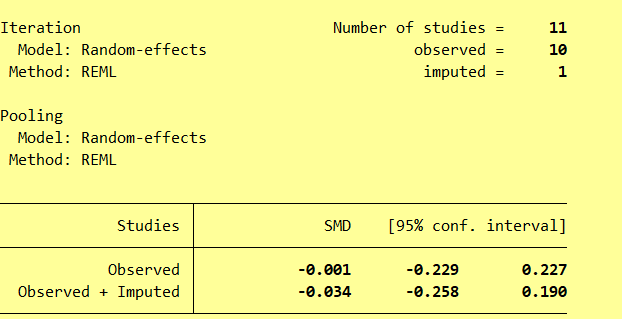
**

**Egger Regression test of FBG**

**
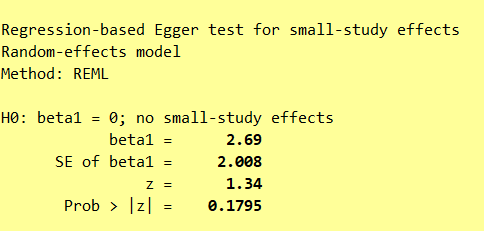
**

**
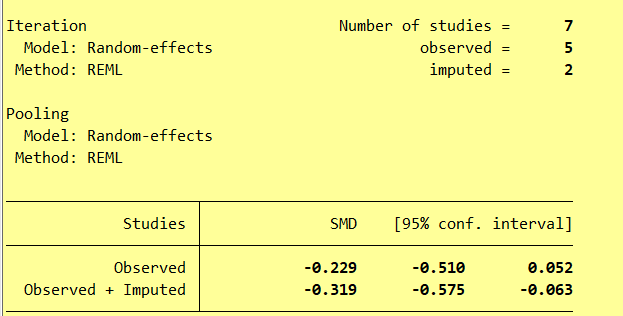
**

**Egger Regression test of HbA1c**

**
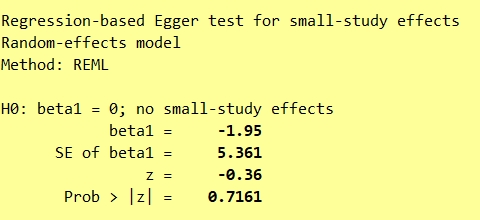
**

**
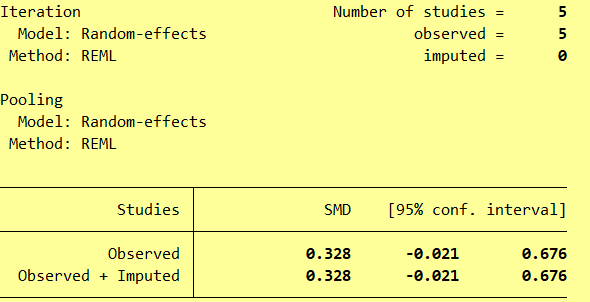
**

**Egger Regression test of Insulin**

**
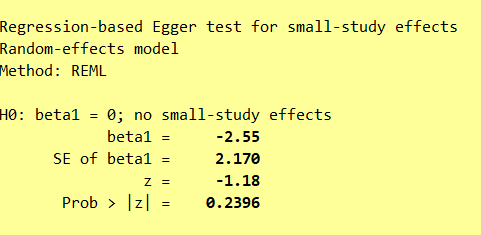
**

**
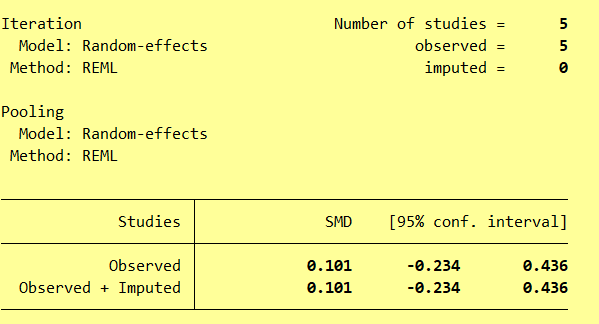
**

**Egger Regression test of TC**

**
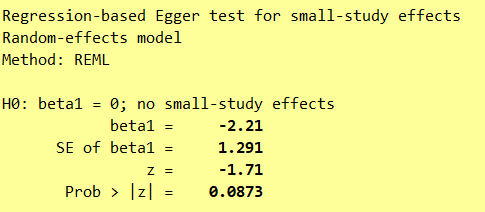
**

**
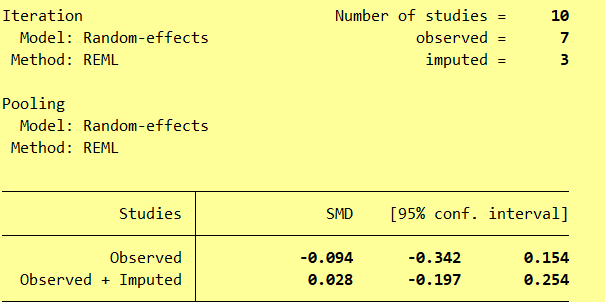
**

**Egger Regression test of TG**

**
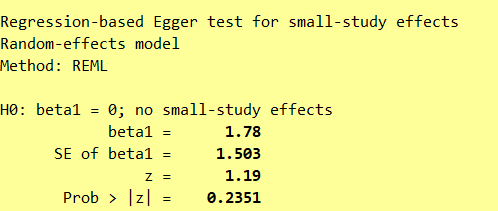
**

**
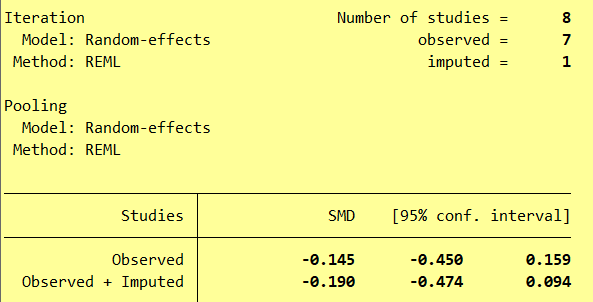
**

**Egger Regression test of LDH**

**
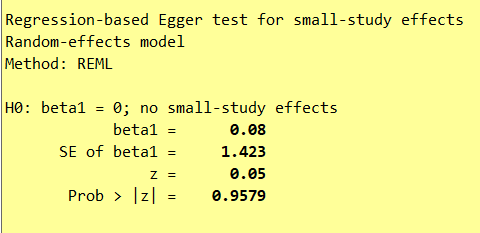
**

**
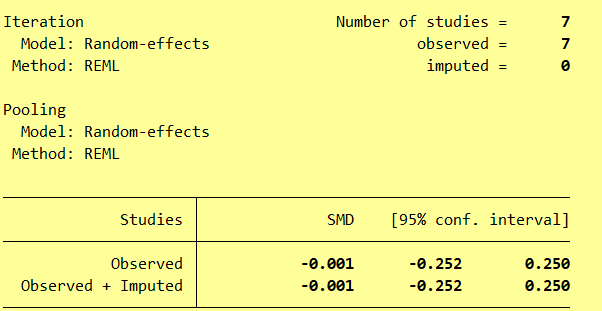
**

**Egger Regression test of LDL**

**
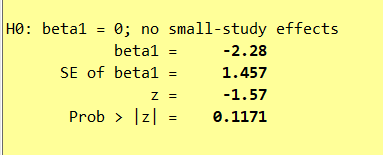
**

**
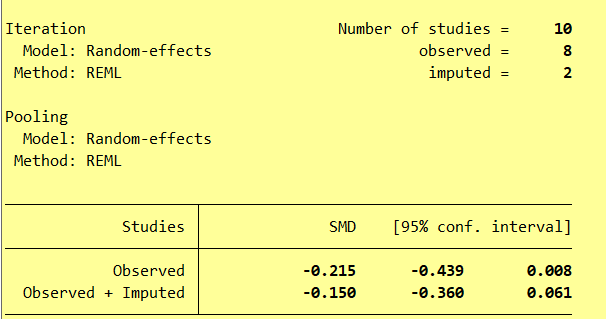
**

**Egger Regression test of systolic BP**

**
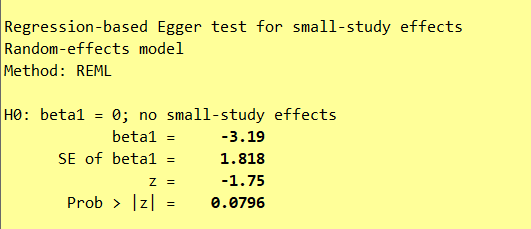
**

**
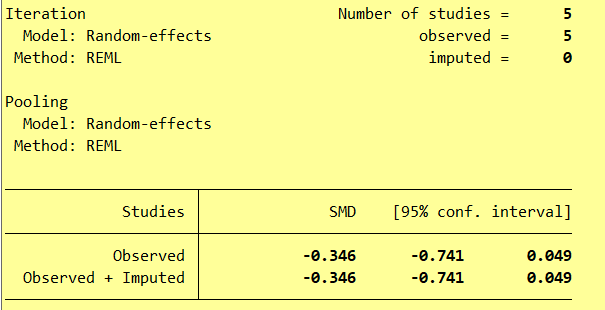
**

**Egger Regression test of diastolic BP**

**
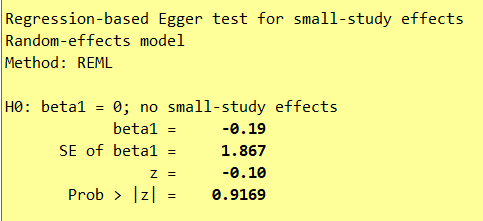
**

**
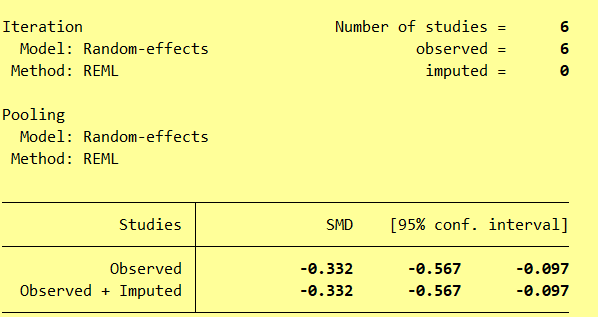
**

**Egger Regression test of CRP**

**
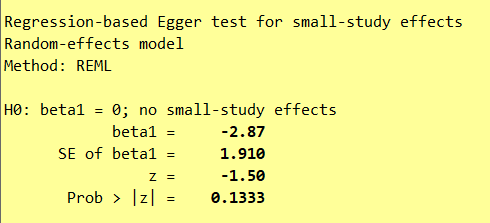
**

**
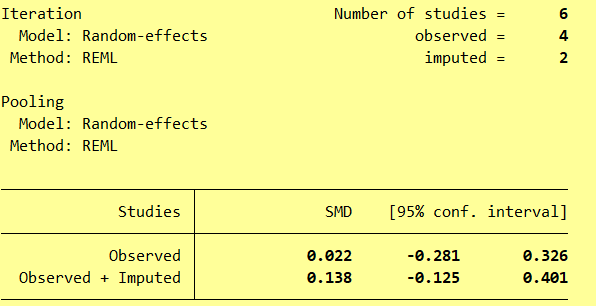
**

**Egger Regression test of IL-6**

**
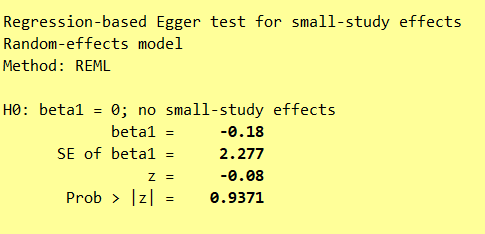
**

**
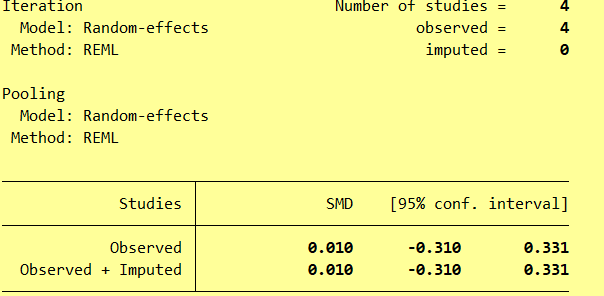
**

**Egger Regression test of TNF-a**
